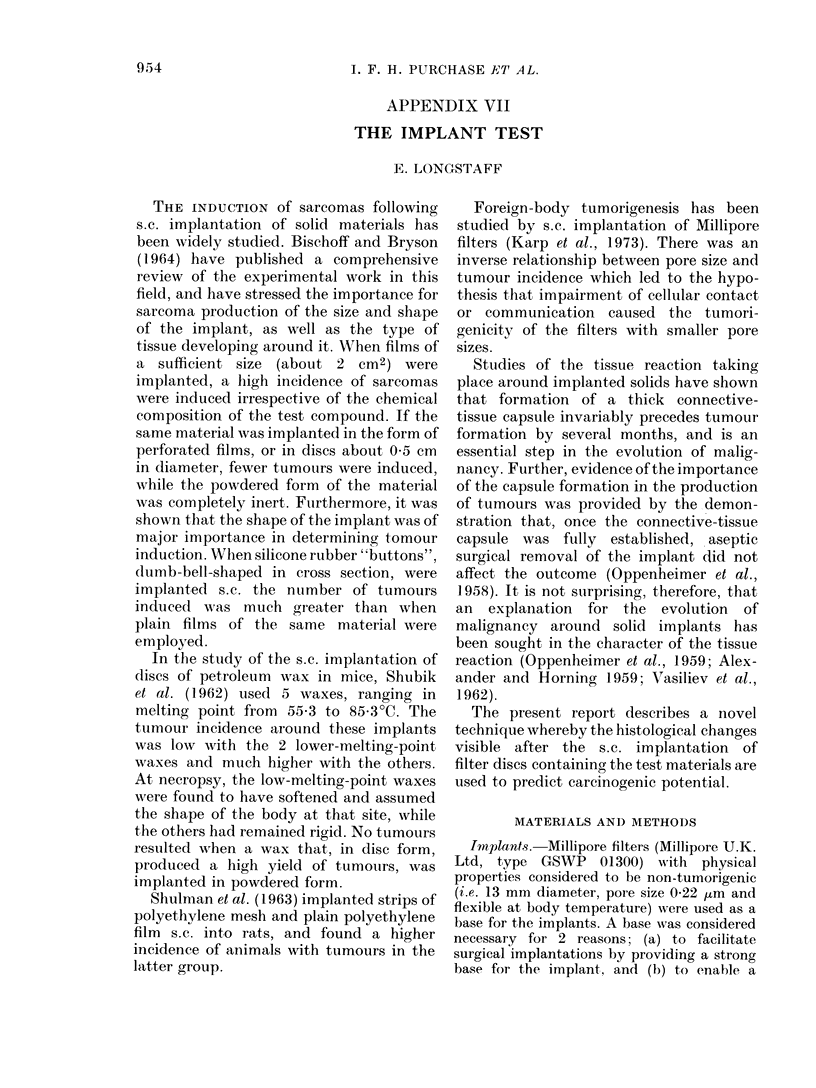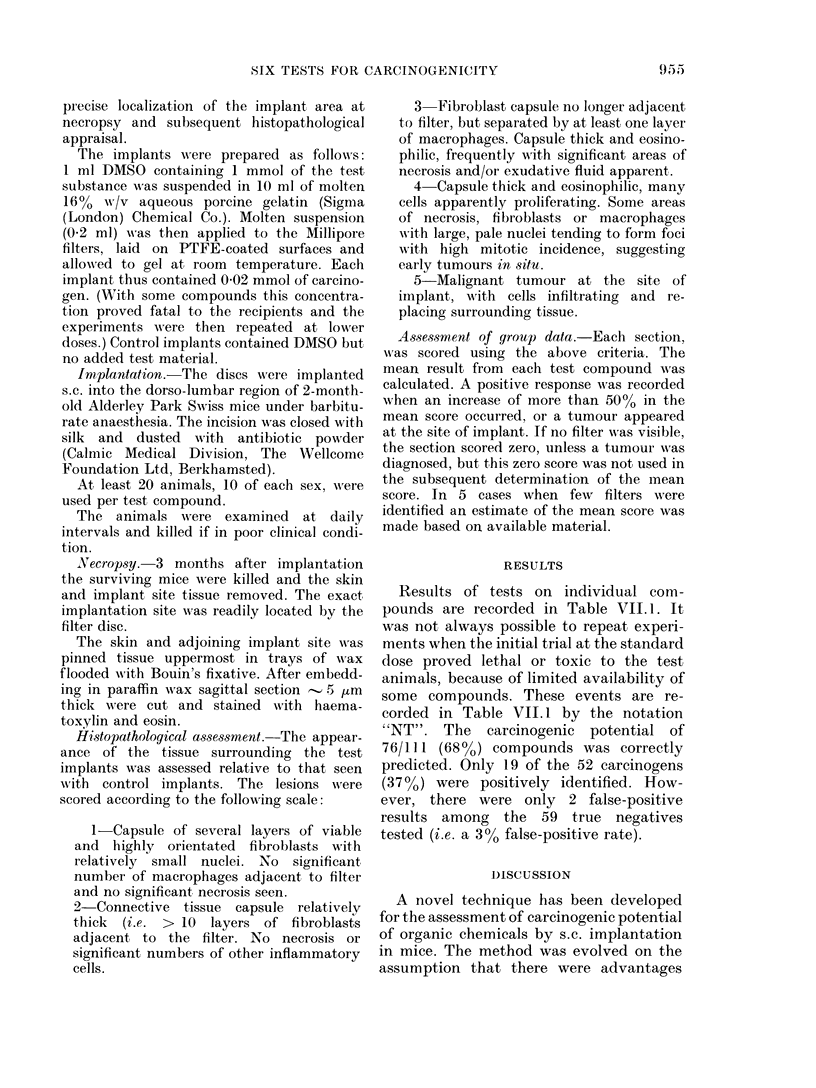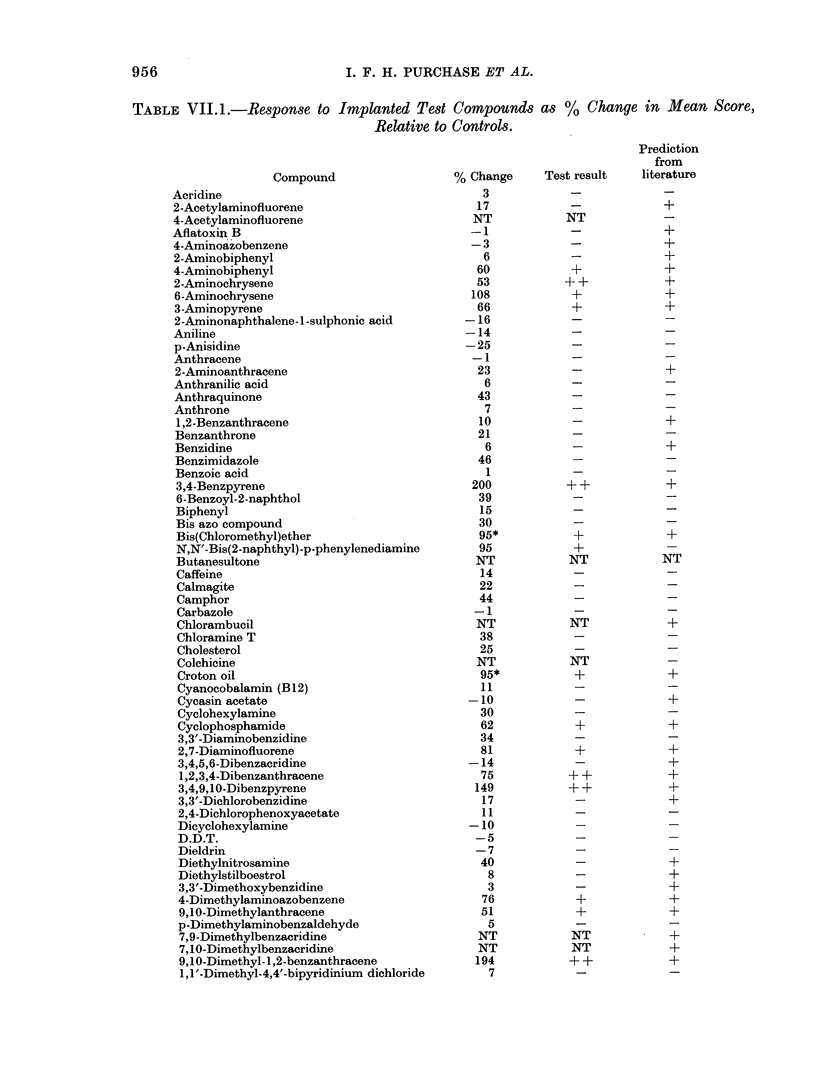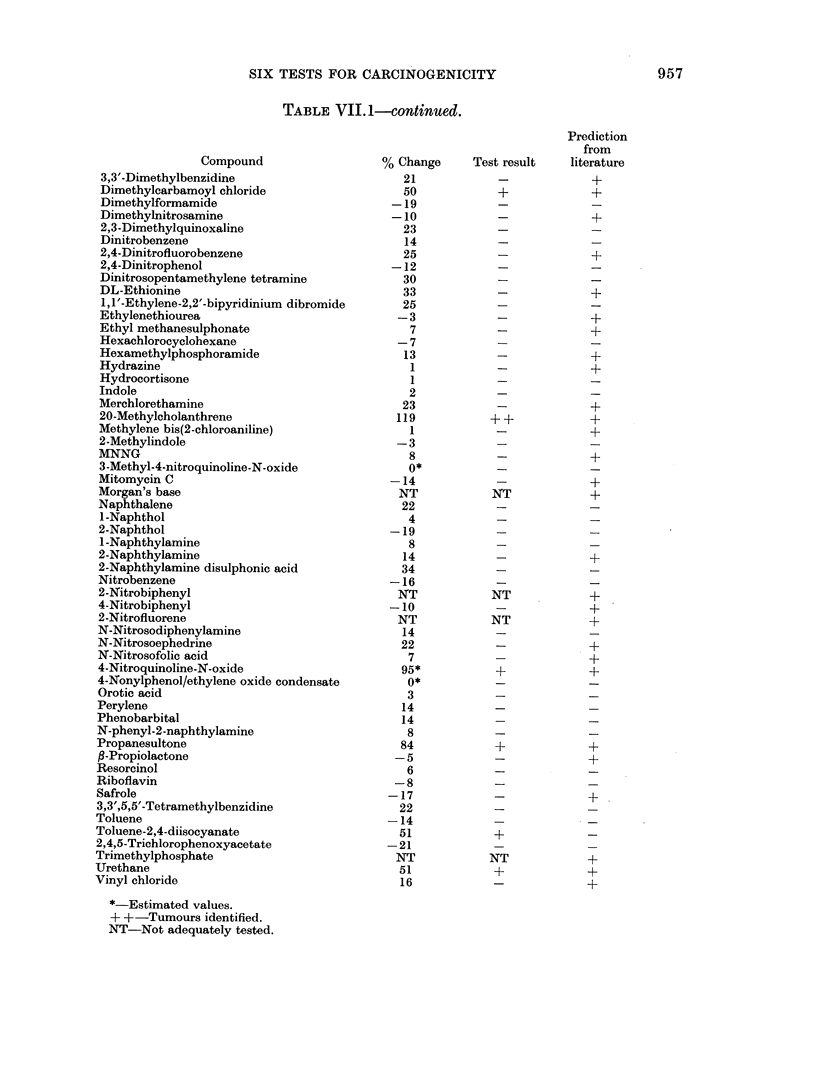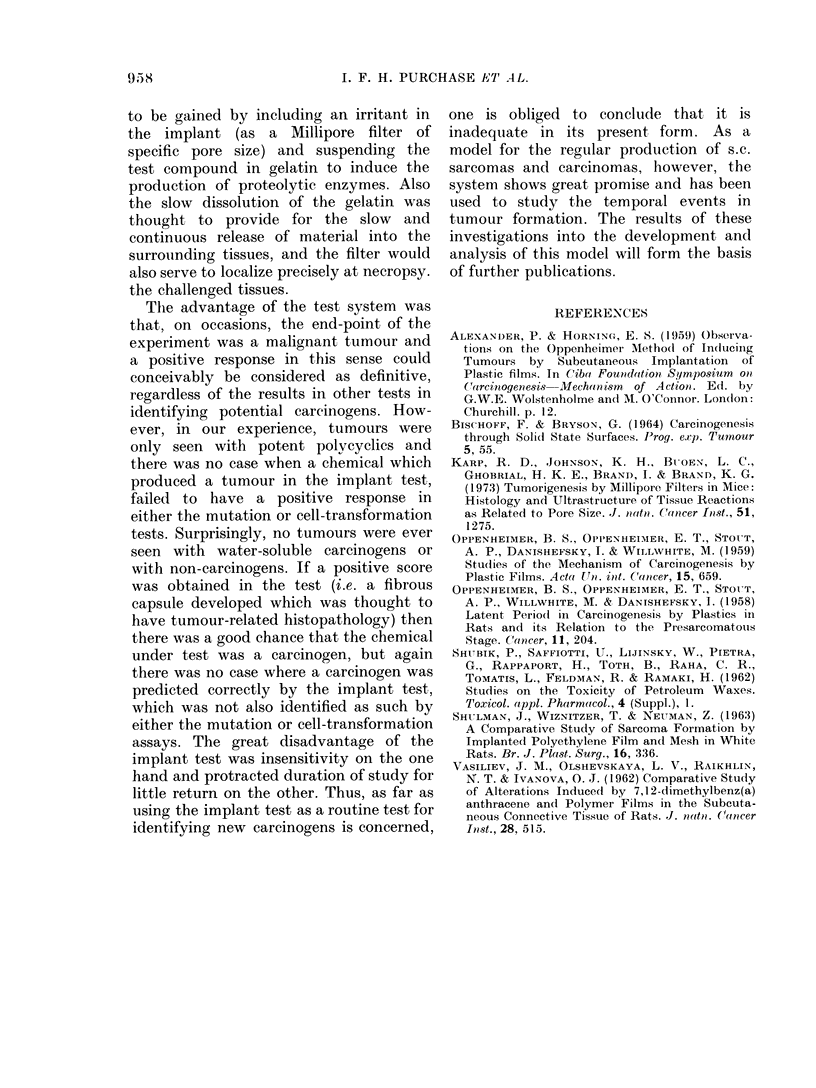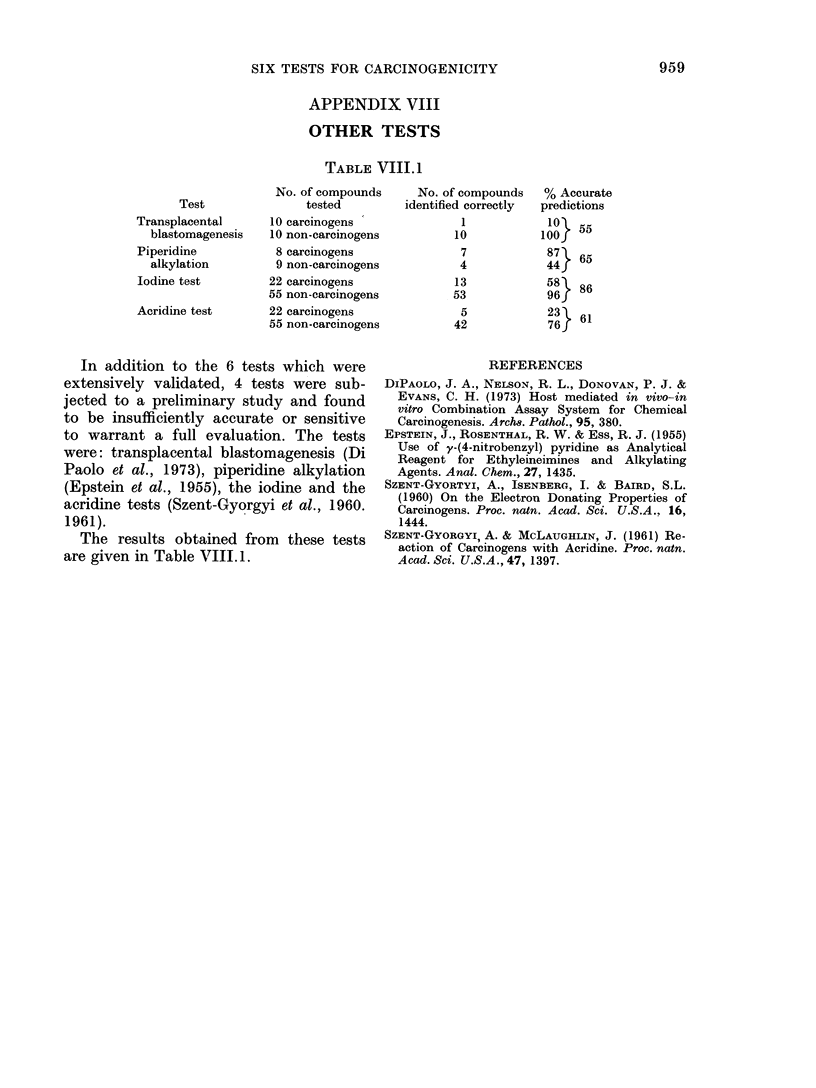# The implant test. Six tests for carcinogenicity.

**DOI:** 10.1038/bjc.1978.139

**Published:** 1978-06

**Authors:** E. Longstaff


### APPENDIX VII

### THE IMPLANT TEST

E. LONGSTAFF

THE INDUCTION of sarcomas following s.c. implantation of solid materials has been widely studied. Bischoff and Bryson (1964) have published a comprehensive review of the experimental work in this field, and have stressed the importance for sarcoma production of the size and shape of the implant, as well as the type of tissue developing around it. When films of a sufficient size (about 2 $cm^2$) were implanted, a high incidence of sarcomas were induced irrespective of the chemical composition of the test compound. If the same material was implanted in the form of perforated films, or in discs about 0·5 cm in diameter, fewer tumours were induced, while the powdered form of the material was completely inert. Furthermore, it was shown that the shape of the implant was of major importance in determining tomour induction. When silicone rubber "buttons", dumb-bell-shaped in cross section, were implanted s.c. the number of tumours induced was much greater than when plain films of the same material were employed.

In the study of the s.c. implantation of discs of petroleum wax in mice, Shubik *et al.* (1962) used 5 waxes, ranging in melting point from 55·3 to 85·3°C. The tumour incidence around these implants was low with the 2 lower-melting-point waxes and much higher with the others. At necropsy, the low-melting-point waxes were found to have softened and assumed the shape of the body at that site, while the others had remained rigid. No tumours resulted when a wax that, in disc form, produced a high yield of tumours, was implanted in powdered form.

Shulman *et al.* (1963) implanted strips of polyethylene mesh and plain polyethylene film s.c. into rats, and found a higher incidence of animals with tumours in the latter group.

Foreign-body tumorigenesis has been studied by s.c. implantation of Millipore filters (Karp *et al.*, 1973). There was an inverse relationship between pore size and tumour incidence which led to the hypothesis that impairment of cellular contact or communication caused the tumorigenicity of the filters with smaller pore sizes.

Studies of the tissue reaction taking place around implanted solids have shown that formation of a thick connective-tissue capsule invariably precedes tumour formation by several months, and is an essential step in the evolution of malignancy. Further, evidence of the importance of the capsule formation in the production of tumours was provided by the demonstration that, once the connective-tissue capsule was fully established, aseptic surgical removal of the implant did not affect the outcome (Oppenheimer *et al.*, 1958). It is not surprising, therefore, that an explanation for the evolution of malignancy around solid implants has been sought in the character of the tissue reaction (Oppenheimer *et al.*, 1959; Alexander and Horning 1959; Vasiliev *et al.*, 1962).

The present report describes a novel technique whereby the histological changes visible after the s.c. implantation of filter discs containing the test materials are used to predict carcinogenic potential.

### MATERIALS AND METHODS

*Implants.*—Millipore filters (Millipore U.K. Ltd, type GSWP 01300) with physical properties considered to be non-tumorigenic (*i.e.* 13 mm diameter, pore size 0·22 μm and flexible at body temperature) were used as a base for the implants. A base was considered necessary for 2 reasons; (a) to facilitate surgical implantations by providing a strong base for the implant, and (b) to enable a precise localization of the implant area at necropsy and subsequent histopathological appraisal.

The implants were prepared as follows: 1 ml DMSO containing 1 mmol of the test substance was suspended in 10 ml of molten 16% w/v aqueous porcine gelatin (Sigma (London) Chemical Co.). Molten suspension (0·2 ml) was then applied to the Millipore filters, laid on PTFE-coated surfaces and allowed to gel at room temperature. Each implant thus contained 0·02 mmol of carcinogen. (With some compounds this concentration proved fatal to the recipients and the experiments were then repeated at lower doses.) Control implants contained DMSO but no added test material.

*Implantation*.—The discs were implanted s.c. into the dorso-lumbar region of 2-month-old Alderley Park Swiss mice under barbiturate anaesthesia. The incision was closed with silk and dusted with antibiotic powder (Calmic Medical Division, The Wellcome Foundation Ltd, Berkhamsted).

At least 20 animals, 10 of each sex, were used per test compound.

The animals were examined at daily intervals and killed if in poor clinical condition.

*Necropsy*.—3 months after implantation the surviving mice were killed and the skin and implant site tissue removed. The exact implantation site was readily located by the filter disc.

The skin and adjoining implant site was pinned tissue uppermost in trays of wax flooded with Bouin's fixative. After embedding in paraffin wax sagittal section $\sim 5\ \mu m$ thick were cut and stained with haematoxylin and eosin.

*Histopathological assessment*.—The appearance of the tissue surrounding the test implants was assessed relative to that seen with control implants. The lesions were scored according to the following scale:

1—Capsule of several layers of viable and highly orientated fibroblasts with relatively small nuclei. No significant number of macrophages adjacent to filter and no significant necrosis seen.

2—Connective tissue capsule relatively thick (*i.e.* $> 10$ layers of fibroblasts adjacent to the filter. No necrosis or significant numbers of other inflammatory cells.

3—Fibroblast capsule no longer adjacent to filter, but separated by at least one layer of macrophages. Capsule thick and eosinophilic, frequently with significant areas of necrosis and/or exudative fluid apparent.

4—Capsule thick and eosinophilic, many cells apparently proliferating. Some areas of necrosis, fibroblasts or macrophages with large, pale nuclei tending to form foci with high mitotic incidence, suggesting early tumours *in situ*.

5—Malignant tumour at the site of implant, with cells infiltrating and replacing surrounding tissue.

*Assessment of group data*.—Each section, was scored using the above criteria. The mean result from each test compound was calculated. A positive response was recorded when an increase of more than 50% in the mean score occurred, or a tumour appeared at the site of implant. If no filter was visible, the section scored zero, unless a tumour was diagnosed, but this zero score was not used in the subsequent determination of the mean score. In 5 cases when few filters were identified an estimate of the mean score was made based on available material.

### RESULTS

Results of tests on individual compounds are recorded in Table VII.1. It was not always possible to repeat experiments when the initial trial at the standard dose proved lethal or toxic to the test animals, because of limited availability of some compounds. These events are recorded in Table VII.1 by the notation "NT". The carcinogenic potential of 76/111 (68%) compounds was correctly predicted. Only 19 of the 52 carcinogens (37%) were positively identified. However, there were only 2 false-positive results among the 59 true negatives tested (*i.e.* a 3% false-positive rate).

### DISCUSSION

A novel technique has been developed for the assessment of carcinogenic potential of organic chemicals by s.c. implantation in mice. The method was evolved on the assumption that there were advantages

TABLE VII.1.—*Response to Implanted Test Compounds as % Change in Mean Score, Relative to Controls.*

| Compound | % Change | Test result | Prediction from literature |
|---|---|---|---|
| Acridine | 3 | − | − |
| 2-Acetylaminofluorene | 17 | − | + |
| 4-Acetylaminofluorene | NT | NT | − |
| Aflatoxin B | −1 | − | + |
| 4-Aminoazobenzene | −3 | − | + |
| 2-Aminobiphenyl | 6 | − | + |
| 4-Aminobiphenyl | 60 | + | + |
| 2-Aminochrysene | 53 | ++ | + |
| 6-Aminochrysene | 108 | + | + |
| 3-Aminopyrene | 66 | + | + |
| 2-Aminonaphthalene-1-sulphonic acid | −16 | − | − |
| Aniline | −14 | − | − |
| p-Anisidine | −25 | − | − |
| Anthracene | −1 | − | − |
| 2-Aminoanthracene | 23 | − | + |
| Anthranilic acid | 6 | − | − |
| Anthraquinone | 43 | − | − |
| Anthrone | 7 | − | − |
| 1,2-Benzanthracene | 10 | − | + |
| Benzanthrone | 21 | − | − |
| Benzidine | 6 | − | + |
| Benzimidazole | 46 | − | − |
| Benzoic acid | 1 | − | − |
| 3,4-Benzpyrene | 200 | ++ | + |
| 6-Benzoyl-2-naphthol | 39 | − | − |
| Biphenyl | 15 | − | − |
| Bis azo compound | 30 | − | − |
| Bis(Chloromethyl)ether | 95* | + | + |
| N,N′-Bis(2-naphthyl)-p-phenylenediamine | 95 | + | − |
| Butanesultone | NT | NT | NT |
| Caffeine | 14 | − | − |
| Calmagite | 22 | − | − |
| Camphor | 44 | − | − |
| Carbazole | −1 | − | − |
| Chlorambucil | NT | NT | + |
| Chloramine T | 38 | − | − |
| Cholesterol | 25 | − | − |
| Colchicine | NT | NT | − |
| Croton oil | 95* | + | + |
| Cyanocobalamin (B12) | 11 | − | − |
| Cycasin acetate | −10 | − | + |
| Cyclohexylamine | 30 | − | − |
| Cyclophosphamide | 62 | + | + |
| 3,3′-Diaminobenzidine | 34 | − | − |
| 2,7-Diaminofluorene | 81 | + | + |
| 3,4,5,6-Dibenzacridine | −14 | − | + |
| 1,2,3,4-Dibenzanthracene | 75 | ++ | + |
| 3,4,9,10-Dibenzpyrene | 149 | ++ | + |
| 3,3′-Dichlorobenzidine | 17 | − | + |
| 2,4-Dichlorophenoxyacetate | 11 | − | − |
| Dicyclohexylamine | −10 | − | − |
| D.D.T. | −5 | − | − |
| Dieldrin | −7 | − | − |
| Diethylnitrosamine | 40 | − | + |
| Diethylstilboestrol | 8 | − | + |
| 3,3′-Dimethoxybenzidine | 3 | − | + |
| 4-Dimethylaminoazobenzene | 76 | + | + |
| 9,10-Dimethylanthracene | 51 | + | + |
| p-Dimethylaminobenzaldehyde | 5 | − | − |
| 7,9-Dimethylbenzacridine | NT | NT | + |
| 7,10-Dimethylbenzacridine | NT | NT | + |
| 9,10-Dimethyl-1,2-benzanthracene | 194 | ++ | + |
| 1,1′-Dimethyl-4,4′-bipyridinium dichloride | 7 | − | − |

TABLE VII.1—*continued*.

| Compound | % Change | Test result | Prediction from literature |
|---|---|---|---|
| 3,3′-Dimethylbenzidine | 21 | − | + |
| Dimethylcarbamoyl chloride | 50 | + | + |
| Dimethylformamide | −19 | − | − |
| Dimethylnitrosamine | −10 | − | + |
| 2,3-Dimethylquinoxaline | 23 | − | − |
| Dinitrobenzene | 14 | − | − |
| 2,4-Dinitrofluorobenzene | 25 | − | + |
| 2,4-Dinitrophenol | −12 | − | − |
| Dinitrosopentamethylene tetramine | 30 | − | − |
| DL-Ethionine | 33 | − | + |
| 1,1′-Ethylene-2,2′-bipyridinium dibromide | 25 | − | − |
| Ethylenethiourea | −3 | − | + |
| Ethyl methanesulphonate | 7 | − | + |
| Hexachlorocyclohexane | −7 | − | − |
| Hexamethylphosphoramide | 13 | − | + |
| Hydrazine | 1 | − | + |
| Hydrocortisone | 1 | − | − |
| Indole | 2 | − | − |
| Merchlorethamine | 23 | − | + |
| 20-Methylcholanthrene | 119 | ++ | + |
| Methylene bis(2-chloroaniline) | 1 | − | + |
| 2-Methylindole | −3 | − | − |
| MNNG | 8 | − | + |
| 3-Methyl-4-nitroquinoline-N-oxide | 0* | − | − |
| Mitomycin C | −14 | − | + |
| Morgan's base | NT | NT | + |
| Naphthalene | 22 | − | − |
| 1-Naphthol | 4 | − | − |
| 2-Naphthol | −19 | − | − |
| 1-Naphthylamine | 8 | − | − |
| 2-Naphthylamine | 14 | − | + |
| 2-Naphthylamine disulphonic acid | 34 | − | − |
| Nitrobenzene | −16 | − | − |
| 2-Nitrobiphenyl | NT | NT | + |
| 4-Nitrobiphenyl | −10 | − | + |
| 2-Nitrofluorene | NT | NT | + |
| N-Nitrosodiphenylamine | 14 | − | − |
| N-Nitrosoephedrine | 22 | − | + |
| N-Nitrosofolic acid | 7 | − | + |
| 4-Nitroquinoline-N-oxide | 95* | + | + |
| 4-Nonylphenol/ethylene oxide condensate | 0* | − | − |
| Orotic acid | 3 | − | − |
| Perylene | 14 | − | − |
| Phenobarbital | 14 | − | − |
| N-phenyl-2-naphthylamine | 8 | − | − |
| Propanesultone | 84 | + | + |
| β-Propiolactone | −5 | − | + |
| Resorcinol | 6 | − | − |
| Riboflavin | −8 | − | − |
| Safrole | −17 | − | + |
| 3,3′,5,5′-Tetramethylbenzidine | 22 | − | − |
| Toluene | −14 | − | − |
| Toluene-2,4-diisocyanate | 51 | + | − |
| 2,4,5-Trichlorophenoxyacetate | −21 | − | − |
| Trimethylphosphate | NT | NT | + |
| Urethane | 51 | + | + |
| Vinyl chloride | 16 | − | + |

*—Estimated values.
++—Tumours identified.
NT—Not adequately tested.

to be gained by including an irritant in the implant (as a Millipore filter of specific pore size) and suspending the test compound in gelatin to induce the production of proteolytic enzymes. Also the slow dissolution of the gelatin was thought to provide for the slow and continuous release of material into the surrounding tissues, and the filter would also serve to localize precisely at necropsy. the challenged tissues.

The advantage of the test system was that, on occasions, the end-point of the experiment was a malignant tumour and a positive response in this sense could conceivably be considered as definitive, regardless of the results in other tests in identifying potential carcinogens. However, in our experience, tumours were only seen with potent polycyclics and there was no case when a chemical which produced a tumour in the implant test, failed to have a positive response in either the mutation or cell-transformation tests. Surprisingly, no tumours were ever seen with water-soluble carcinogens or with non-carcinogens. If a positive score was obtained in the test (*i.e.* a fibrous capsule developed which was thought to have tumour-related histopathology) then there was a good chance that the chemical under test was a carcinogen, but again there was no case where a carcinogen was predicted correctly by the implant test, which was not also identified as such by either the mutation or cell-transformation assays. The great disadvantage of the implant test was insensitivity on the one hand and protracted duration of study for little return on the other. Thus, as far as using the implant test as a routine test for identifying new carcinogens is concerned, one is obliged to conclude that it is inadequate in its present form. As a model for the regular production of s.c. sarcomas and carcinomas, however, the system shows great promise and has been used to study the temporal events in tumour formation. The results of these investigations into the development and analysis of this model will form the basis of further publications.

### REFERENCES

Alexander, P. & Horning, E. S. (1959) Observations on the Oppenheimer Method of Inducing Tumours by Subcutaneous Implantation of Plastic films. In *Ciba Foundation Symposium on Carcinogenesis—Mechanism of Action.* Ed. by G.W.E. Wolstenholme and M. O'Connor. London: Churchill. p. 12.

Bischoff, F. & Bryson, G. (1964) Carcinogenesis through Solid State Surfaces. *Prog. exp. Tumour* **5**, 55.

Karp, R. D., Johnson, K. H., Buoen, L. C., Ghobrial, H. K. E., Brand, I. & Brand, K. G. (1973) Tumorigenesis by Millipore Filters in Mice: Histology and Ultrastructure of Tissue Reactions as Related to Pore Size. *J. natn. Cancer Inst.*, **51**, 1275.

Oppenheimer, B. S., Oppenheimer, E. T., Stout, A. P., Danishefsky, I. & Willwhite, M. (1959) Studies of the Mechanism of Carcinogenesis by Plastic Films. *Acta Un. int. Cancer*, **15**, 659.

Oppenheimer, B. S., Oppenheimer, E. T., Stout, A. P., Willwhite, M. & Danishefsky, I. (1958) Latent Period in Carcinogenesis by Plastics in Rats and its Relation to the Presarcomatous Stage. *Cancer*, **11**, 204.

Shubik, P., Saffiotti, U., Lijinsky, W., Pietra, G., Rappaport, H., Toth, B., Raha, C. R., Tomatis, L., Feldman, R. & Ramaki, H. (1962) Studies on the Toxicity of Petroleum Waxes. *Toxicol. appl. Pharmacol.*, **4** (Suppl.), 1.

Shulman, J., Wiznitzer, T. & Neuman, Z. (1963) A Comparative Study of Sarcoma Formation by Implanted Polyethylene Film and Mesh in White Rats. *Br. J. Plast. Surg.*, **16**, 336.

Vasiliev, J. M., Olshevskaya, L. V., Raikhlin, N. T. & Ivanova, O. J. (1962) Comparative Study of Alterations Induced by 7,12-dimethylbenz(a) anthracene and Polymer Films in the Subcutaneous Connective Tissue of Rats. *J. natn. Cancer Inst.*, **28**, 515.

### APPENDIX VIII

### OTHER TESTS

TABLE VIII.1

| Test | No. of compounds tested | No. of compounds identified correctly | % Accurate predictions | |
|---|---|---|---|---|
| Transplacental blastomagenesis | 10 carcinogens | 1 | 10 | 55 |
| | 10 non-carcinogens | 10 | 100 | |
| Piperidine alkylation | 8 carcinogens | 7 | 87 | 65 |
| | 9 non-carcinogens | 4 | 44 | |
| Iodine test | 22 carcinogens | 13 | 58 | 86 |
| | 55 non-carcinogens | 53 | 96 | |
| Acridine test | 22 carcinogens | 5 | 23 | 61 |
| | 55 non-carcinogens | 42 | 76 | |

In addition to the 6 tests which were extensively validated, 4 tests were subjected to a preliminary study and found to be insufficiently accurate or sensitive to warrant a full evaluation. The tests were: transplacental blastomagenesis (Di Paolo *et al.*, 1973), piperidine alkylation (Epstein *et al.*, 1955), the iodine and the acridine tests (Szent-Gyorgyi *et al.*, 1960. 1961).

The results obtained from these tests are given in Table VIII.1.

### REFERENCES

DIPAOLO, J. A., NELSON, R. L., DONOVAN, P. J. & EVANS, C. H. (1973) Host mediated *in vivo-in vitro* Combination Assay System for Chemical Carcinogenesis. *Archs. Pathol.*, **95**, 380.

EPSTEIN, J., ROSENTHAL, R. W. & ESS, R. J. (1955) Use of $\gamma$-(4-nitrobenzyl) pyridine as Analytical Reagent for Ethyleineimines and Alkylating Agents. *Anal. Chem.*, **27**, 1435.

SZENT-GYORTYI, A., ISENBERG, I. & BAIRD, S.L. (1960) On the Electron Donating Properties of Carcinogens. *Proc. natn. Acad. Sci. U.S.A.*, **16**, 1444.

SZENT-GYORGYI, A. & MCLAUGHLIN, J. (1961) Reaction of Carcinogens with Acridine. *Proc. natn. Acad. Sci. U.S.A.*, **47**, 1397.